# Pathological Association of Coracoacromial Arch Tendons in Shoulder Impingement

**DOI:** 10.7759/cureus.113536

**Published:** 2026-07-28

**Authors:** Amr Elmaraghy, Humood Boqambar, Flo Kim, Amanda Pennings, Patrick Carroll

**Affiliations:** 1 Orthopaedic Surgery, St. Joseph’s Health Centre, Unity Health Toronto, Toronto, CAN; 2 Surgery, Li Ka Shing Knowledge Institute, Toronto, CAN; 3 Surgery, University of Toronto, Toronto, CAN; 4 Surgery, St. Joseph’s Health Centre, Unity Health Toronto, Toronto, CAN; 5 Family and Community Medicine, University of Toronto, Toronto, CAN

**Keywords:** long head of biceps tendon, shoulder arthroscopy, shoulder impingement, subscapularis tendon, supraspinatus tendon

## Abstract

Background: Pathology of the supraspinatus, subscapularis, and long head of biceps (LHB) can occur concurrently with shoulder impingement syndrome. Our study investigated the incidence and association of structurally significant pathology in these three tendons in subacromial impingement.

Methods: A retrospective cohort of 250 shoulders of 240 patients with subacromial impingement syndrome that underwent arthroscopic subacromial decompression after failing three to six months of conservative management between November 2008 and December 2011 was included.

Results: Structurally significant pathology was present in the supraspinatus tendon in 124 (49.6%) shoulders, the LHB tendon in 42 (16.8%) shoulders, and the subscapularis tendon in 45 (18%) shoulders. Of those with supraspinatus pathology, 24.2% had LHB pathology, and 27.4% had subscapularis pathology. Structurally significant supraspinatus pathology was associated with LHB and/or subscapularis pathology in 39.5% of shoulders. Patients with structural pathology of all three tendons were significantly older (57.2 ± 7.8, P = 0.004), more likely to be male (P = 0.006), and have the dominant arm affected (P = 0.037).

Conclusion: Pathology of supraspinatus, LHB, and subscapularis tendons often occurs concomitantly in subacromial impingement. This study highlights this intimate relationship and serves as a clinical reminder for thorough evaluation of all three tendons to ensure complete diagnosis and treatment.

## Introduction

The supraspinatus, long head of biceps (LHB), and subscapularis tendons are three distinct yet anatomically and functionally interconnected structures that often demonstrate concurrent pathology in patients with shoulder subacromial impingement. These tendons are situated in close proximity under the coracoacromial arch and span the greater and lesser tuberosities and the intertubercular groove of the proximal humerus.

Subacromial impingement syndrome is a common diagnosis of shoulder pain and dysfunction. The diagnosis of shoulder subacromial impingement includes varying degrees of pathology, including subacromial bursitis, LHB tendinopathy and/or instability, rotator cuff tendinopathy and/or rotator cuff tears [1]. In subacromial impingement, the tendons of the rotator cuff, most commonly the supraspinatus tendon, become compressed between the humeral head below and the coracoacromial arch above [1]. Clinical experience and anatomical studies have suggested that pathology often extends to the LHB and subscapularis tendons. Multiple studies have examined concomitant pathology of the biceps tendon and rotator cuff, suggesting that supraspinatus pathology should be considered within the broader context of rotator cuff and biceps involvement. These findings highlight the diagnostic relevance of evaluating all three tendons together when assessing subacromial impingement [2-7]. However, these associated lesions may go undetected without deliberate evaluation, both preoperatively and intraoperatively. Few studies have specifically examined the incidence and patterns of concurrent involvement of the supraspinatus, LHB, and subscapularis tendons [2,3,7,8]. This gap in the literature provides the rationale for investigating how these tendons may be pathologically associated in patients with subacromial impingement. The clinical value of recognizing and evaluating all three tendons is substantial. Missed pathology of one or more of these structures during surgery may result in incomplete treatment and persistent symptoms [9]. This highlights the need for heightened clinical awareness and systematic examination - clinically, radiologically, and arthroscopically - of all three tendons.

The development of shoulder arthroscopy has allowed shoulder surgeons to diagnose and treat shoulder pathology in a minimally invasive manner. Shoulder arthroscopy has shown benefit over clinical and radiological investigations preoperatively and - although user dependent - has the potential to provide a more accurate diagnosis [10].

The main objective of this study was to determine the prevalence and patterns of concomitant supraspinatus, LHB, and subscapularis tendon pathology identified during arthroscopy in patients undergoing surgery for subacromial impingement. Our hypothesis was that concomitant lesions of the supraspinatus, LHB, and subscapularis tendons would occur frequently in a series of patients with subacromial impingement syndrome.

## Materials and methods

Ethics approval was obtained from the St. Joseph’s Health Centre Research Ethics Board (approval no. 2012-011E). A retrospective chart review of all consecutive patients with a diagnosis of subacromial impingement who underwent arthroscopic subacromial decompression between November 2008 and December 2011 was conducted. Clinical diagnosis was established through a standardized history and physical examination, including bilateral range-of-motion measurements; assessment of rotator cuff integrity using the Empty Can (Jobe) test [11] and Belly Press test [12]; evaluation of the LHB tendon using Speed's test [13] and Yergason's test [14]; assessment for subacromial impingement using the Hawkins-Kennedy test [15] and Neer impingement sign [16]; and evaluation of glenohumeral instability using the Apprehension-Relocation test [17]. Findings from the physical exam were correlated with plain radiographs and magnetic resonance imaging (MRI) for all patients. Inclusion criteria consisted of patients with (1) clinical and radiological diagnosis of subacromial impingement and (2) who failed conservative treatment and underwent any combination of the following concomitant procedures: arthroscopic rotator cuff repair, subcoracoid decompression, capsular release, debridement, and distal clavicle excision. Patients were excluded if they (1) had a diagnosis of glenohumeral instability requiring labral repair or stabilization, (2) had osteoarthritis (greater than Kellgren and Lawrence grade II) [18], (3) had a previous fracture of the affected shoulder, (4) had undergone previous surgery of the affected shoulder, (5) underwent a revision procedure, and/or (6) underwent open rotator cuff repair. Patients were also excluded if operative and/or consult notes were not available or incomplete. Patients underwent conservative management if they had a negative full-thickness tear on imaging or had a full-thickness tear but elected for non-operative treatment. Conservative measures included physical therapy and possible injection for three to six months. Failure of conservative treatment was indicated by ongoing symptoms.

All surgeries were performed by the same surgeon at our institution under general anesthesia with an inter-scalene block for post-operative pain control. Patients were positioned in a beach chair with a custom traction device set up that included the operative arm in a shoulder positioner with an axillary roll to enhance intraoperative joint visualization and surgical accessibility [19]. Both the bursal and the articular extent of the supraspinatus and subscapularis tendons, as well as both the intra-articular and groove portions of the LHB tendon, were thoroughly examined as part of an initial diagnostic arthroscopy. Patients then underwent arthroscopic surgical treatment for the identified and confirmed rotator cuff pathology, along with other concomitant procedures as necessary. Patient demographic information, preoperative and intraoperative findings, and details of the surgical procedure were recorded, as were lesions to the supraspinatus, LHB, and subscapularis tendons. The number of tendons involved and the distribution of tendon involvement were recorded after being definitively documented intraoperatively. Rotator cuff pathology was characterized in terms of depth of involvement for each tendon and categorized as either no significant structural pathology (normal, tendinopathy without tear, or low-grade partial-thickness tear), or significant structural pathology (moderate-grade partial-thickness tear, high-grade partial-thickness tear, or full-thickness tear). High-grade tears were defined as those involving greater than 50% of the tendon thickness, and low-grade tears were those involving less than 50% [20].

LHB pathology was characterized in terms of stability and depth of involvement and categorized as no significant structural pathology (stable and either normal, fraying, tenosynovitis, erythema, tendinopathy without tear or low-grade partial-thickness tear) or significant structural pathology (unstable and/or either moderate-grade partial-thickness tear, high-grade partial-thickness tear or full-thickness tear).

Data were analyzed using IBM SPSS Statistics for Windows, Version 21 (Released 2012; IBM Corp., Armonk, New York, United States). In cases where bilateral shoulders were involved, each shoulder was handled statistically as a unique entry. Data analysis included frequencies and percentages for discrete data and mean and standard deviation for continuous data. Categorical variables were analysed using the chi-square test, whereas continuous variables were analysed using the independent-samples t-test. Data normality was assessed using the Shapiro-Wilk test. A two-sided P-value of <0.05 was considered statistically significant.

## Results

Arthroscopic subacromial decompressions were performed on 287 shoulders from November 2008 to December 2011. Thirty-seven of these cases were removed based on the exclusion criteria. The remaining 250 shoulders of 240 patients were included in the study (Table 1).

**Table 1:** Patient Demographic Data SD: standard deviation; ^a^ Pathology is structurally significant.

	All Shoulders (N = 250)	Supraspinatus Pathology^a^
Number of shoulders	250	124
Number of subjects	240	122
Age in years (mean ± SD)	51.0 ± 8.6	54.2 ± 7.9
Sex, n (%)		
Male	148 (59.2)	78 (62.9)
Female	102 (40.8)	46 (37.1)
Dominant arm, n (%)		
Right	237 (94.8)	117 (94.4)
Left	13 (5.2)	7 (5.6)
Affected arm, n (%)		
Right	150 (60.0)	81 (65.3)
Left	100 (40.0)	43 (34.7)
History of smoking, n (%)		
Yes	52 (20.8)	21 (16.9)
No	198 (79.2)	103 (83.1)
Mechanism of injury, n (%)		
Trauma	119 (47.6)	67 (54.0)
Subacute	131 (52.4)	57 (46.0)

All patients who underwent surgery had preoperative MRI. The mean age of patients was 51.0 ± 8.6 years (range, 24 to 74 years) at the time of surgery. There were 148 (59.2%) male patients and 102 (40.8%) female patients. Surgical procedures performed are summarized in Table 2.

**Table 2:** Surgical Procedures Performed

Surgical Procedures	Number (N = 250)	Percentage (%)
Subacromial decompression	250	100
Rotator cuff repair	130	52.0
Acromioclavicular joint excision	59	23.6
Biceps tenotomy	46	18.4
Subcoracoid decompression	32	12.8
Biceps tenodesis	5	2.0

The age of patients with structural pathology of all three tendons was 57.2 ± 7.8 (t(248) = 2.929, P = 0.004), which was significantly higher than that of patients without involvement of all three tendons. Patients with structural pathology of all three tendons were also significantly more likely to be male (χ^2^ = 7.697, P = 0.006) and have the dominant arm affected (χ^2^ = 4.358, P = 0.037) (Table 3).

**Table 3:** Clinical Characteristics of Patients With Three Tendon Pathology SD: standard deviation; χ^2^: chi-square test; t: independent samples t-test

Variable	Finding (Three Tendon Pathology vs. Without)	Test Statistic	P-value
Age (years, mean ± SD)	57.2 ± 7.8	t(248) = 2.929	0.004
Sex	Significantly more likely to be male	χ^2^ = 7.697	0.006
Affected arm	Significantly more likely to be the dominant arm	χ^2^ = 4.358	0.037

One hundred forty-four patients (57.6%) had structurally significant pathology of at least one of the three tendons. Structurally significant pathology was present in the supraspinatus tendon in 49.6% (124 patients), in the LHB tendon in 16.8% (42 patients), and in the subscapularis tendon in 18.0% (45 patients) (Table 4).

**Table 4:** Pathology of the Three Tendons n/a: not applicable; PT: partial-thickness. Chi-square tests were applied to categorical variables.

Tendon Pathology	Supraspinatus, n (%)	Long Head of Biceps, n (%)	Subscapularis, n (%)
Structurally Significant Pathology	124 (49.6)	42 (16.8)	45 (18.0)
Full-Thickness Tear	107 (42.8)	20 (8.0)	25 (10.0)
Moderate- to High-Grade PT	17 (6.8)	4 (1.6)	20 (8.0)
Instability	n/a	18 (7.2)	n/a
No Structurally Significant Pathology	126 (50.4)	208 (83.2)	205 (82.0)
Normal	44 (17.6)	138 (55.2)	162 (64.8)
Tendinopathy/Low-Grade PT	82 (32.8)	70 (28.0)	43 (17.2)
Concomitant Tendon Pathology			
Supraspinatus	n/a	P = 0.002	P = 0.000
Long Head of Biceps	P = 0.002	n/a	P = 0.000
Subscapularis	P = 0.000	P = 0.000	n/a

**Figure 1:**
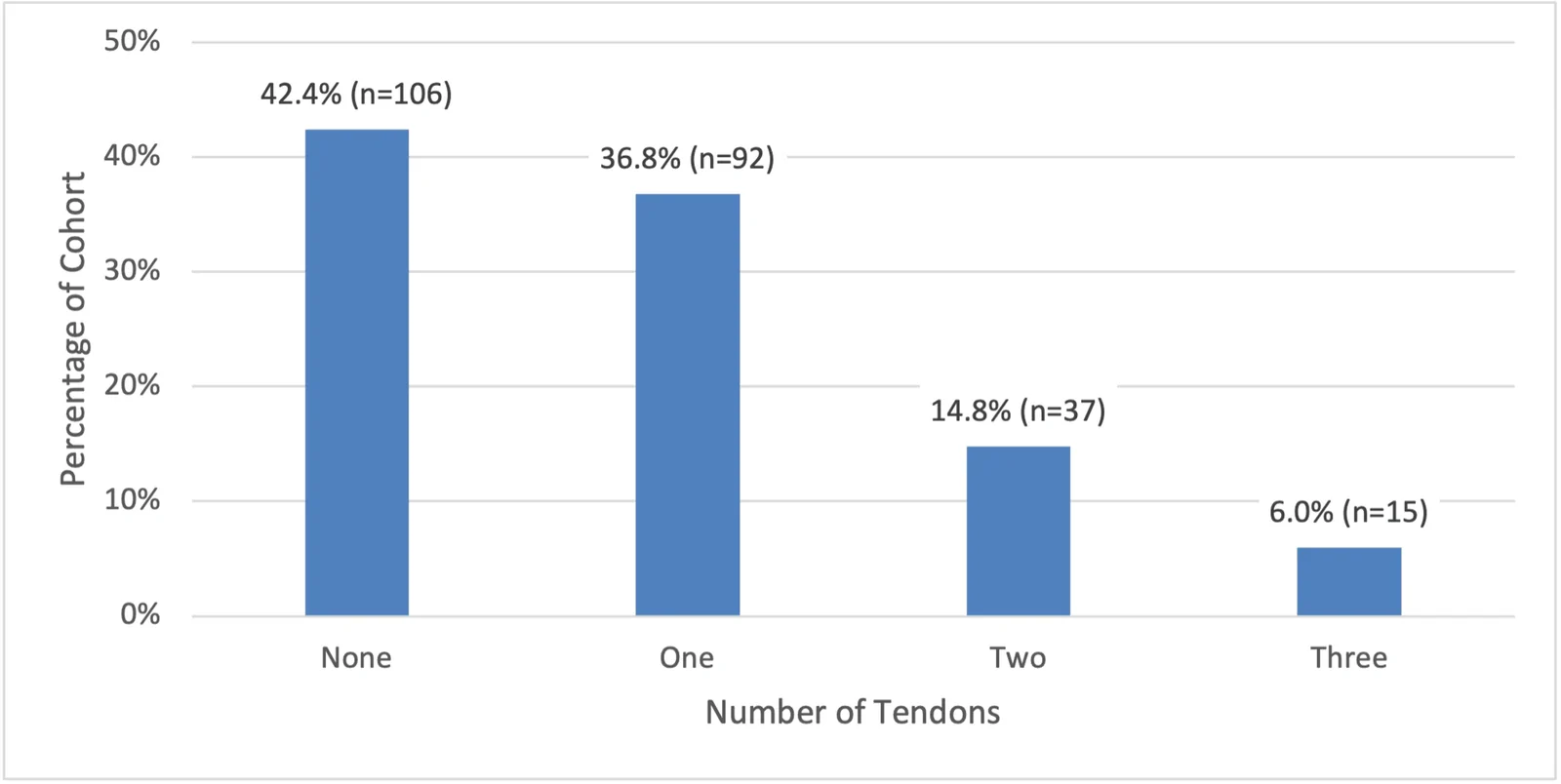
Distribution and Patterns of Tendon Pathology

There were significant associations between pathology of the supraspinatus, LHB, and subscapularis (Table 4). Ninety-two patients (36.8%) had structurally significant pathology of one of the three tendons, 14.8% (37 patients) with pathology of two of the three tendons, and 6.0% (15 patients) with pathology of all three tendons. In cases where none of the three tendons had significant structural pathology (42.4%), either no clinically significant damage to any of these tendons was found on arthroscopic exploration or another rotator cuff tendon was pathological (Figure 1).

Pathology of the LHB and subscapularis was also analyzed in the presence of supraspinatus pathology. One hundred twenty-four patients (49.6%) had structurally significant pathology of the supraspinatus tendon. There were 78 males (62.9%) and 46 females (37.1%) with an average age of 54.2 ± 7.9 (range: 35-74). Of the 124 patients with structurally significant pathology of the supraspinatus tendon, 24.2% (30 patients) had pathology of the LHB tendon, and 27.4% (34 patients) had pathology of the subscapularis tendon. A total of 39.5% of patients who had structurally significant pathology of the supraspinatus also had structurally significant pathology of at least one additional tendon (Table 5).

**Table 5:** Characteristics of Patients With Structurally Significant Supraspinatus Pathology SD: standard deviation

Variable	Supraspinatus Pathology (n = 124)
Demographics	
Age (Years, Mean ± SD)	54.2 ± 7.9
Age Range (Years)	35-74
Male Sex, n (%)	78 (62.9)
Female Sex, n (%)	46 (37.1)
Concomitant Tendon Pathology	
Isolated Supraspinatus Pathology (0 Additional Tendons), n (%)	75 (60.5)
≥1 Additional Tendon Involved, n (%)	49 (39.5)
Involving Long Head of Biceps, n (%)	30 (24.2)
Involving Subscapularis, n (%)	34 (27.4)
Involving Both (Three Tendon Pathology), n (%)	15 (12.1)

## Discussion

This study investigated the frequency and association of structural pathology involving the supraspinatus, LHB, and subscapularis tendons in patients undergoing arthroscopic treatment for subacromial impingement. We suggest that when one of the three tendons has significant structural pathology, it is imperative that this pathology is not focused upon at the exclusion of a thorough preoperative and intraoperative diagnostic examination of the other two tendons.

Our results supported the hypothesis that in a consecutive series of patients with subacromial impingement syndrome, concomitant lesions of the supraspinatus, LHB, and subscapularis tendons occur quite frequently. We found that significant pathology was present in more than one of the three tendons in approximately 21% of patients with shoulder subacromial impingement. The fact that clinical focus is often on the effect of subacromial impingement on the supraspinatus is supported by the predominance of its involvement in our study. However, in the presence of structurally significant pathology of the supraspinatus, 39% of patients also had a combination of LHB and/or subscapularis tendon pathology. Other authors have found that patients often present with concomitant rotator cuff and/or LHB pathology, although patterns of tendon involvement have not been previously explored [2-4,7,8].

Little information is available in the literature regarding the frequency of LHB and subscapularis tendon pathology in the presence of supraspinatus pathology [3,21]. As supraspinatus pathology is well-established and is frequently diagnosed with subacromial impingement, the incidence of associated tendon involvement is clinically relevant for all shoulder surgeons. With such a high frequency of multiple tendon involvement in shoulder pathology, we recommend that examination of all three tendons be a routine part of diagnostic arthroscopy. Additionally, pathology confirmed preoperatively should not distract from particular attention to the neighbouring three tendons. These results reinforce the clinical relevance of a comprehensive diagnostic and surgical approach.

**Figure 2:**
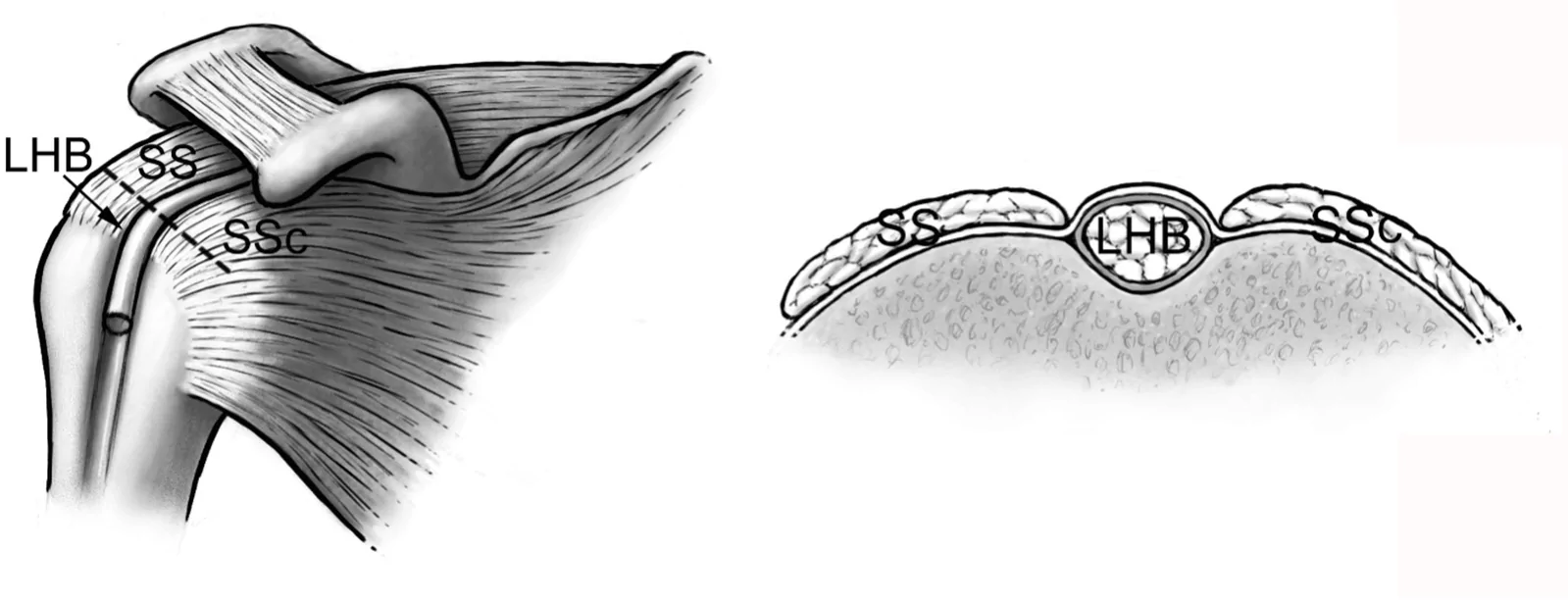
Oblique Cross-Section of the Proximal Humerus in the Plane of the Coracoacromial Arch This illustration depicts the anatomical region where the supraspinatus (SS), long head of the biceps (LHB), and subscapularis (SSc) tendons converge and are susceptible to subacromial impingement, creating the characteristic anatomical "Three Sisters" appearance. Image credits: Dr. Amr Elmaraghy. No AI tools were used to generate or modify the image.

The anatomical proximity and mechanical interplay among these three tendons offer an explanation of their frequent co-involvement. These three anatomical structures are similarly susceptible to the effects of subacromial impingement because of their close anatomical proximity, often likened to the "Three Sisters" mountain range (Figure 2). In addition to the three tendons' shared proximity to the offending coracoacromial arch, several studies have shown that there is a direct anatomical relationship between these three tendons. The supraspinatus and subscapularis insert at the greater and lesser tuberosities, respectively, with the LHB coursing between them in the bicipital groove. An extension of the supraspinatus tendon is interwoven with fibers of the subscapularis tendon and extends across the bicipital groove [7,22,23]. It has been suggested that flattening of the LHB tendon or increased stress on the subscapularis due to a supraspinatus tear may cause an incomplete tear of the subscapularis tendon, further demonstrating that pathology of the supraspinatus, LHB, and subscapularis tendons may be frequently associated [7].

Since even high-quality imaging can be misleading, it should not be relied upon in isolation to evaluate the three tendons. MRI has been shown to be useful for diagnosing supraspinatus lesions, but can often miss subscapularis or LHB tendon pathology [2,24,25]. Even with MRI results, pathology of the LHB and subscapularis tendons may be difficult to diagnose clinically, and the extent of the lesion may be difficult to assess surgically [4,6,7,26]. A study by Garavaglia et al. showed that a greater frequency of subscapularis tears was identified arthroscopically as opposed to the documented MRI results [24]. Thus, pathology of the subscapularis and LHB tendons may be missed without a high index of suspicion and purposeful physical examination, appropriate diagnostic imaging, and thorough arthroscopic evaluation of these tissues. Failure to identify these lesions has been associated in prior studies with persistent symptoms, underscoring the diagnostic importance of evaluating all components of the coraco‑acromial arch [3,27].

The frequency of structurally significant pathology of the supraspinatus (49.6%) and subscapularis (18%) in the present study was substantially less than that reported in a study by Garavaglia et al. [24], who observed supraspinatus tears in 89% and subscapularis tears in 37% of cases. However, unlike our approach, that study did not differentiate between partial-thickness and full-thickness tears. When all tendon pathology - ranging from tendinopathy to full-thickness tears - is included, the overall frequency in the present study aligns closely with their findings [24]. Similarly, Beall et al. reported that 20.7% of patients (23 of 111) had partial- or full-thickness LHB tendon tears [2], which compares to 16.8% (42 of 250) with significant structural pathology found intraoperatively in our cohort. This slight difference may stem from the fact that Beall et al. [2] did not grade partial-thickness tears as low or high grade, likely incorporating low-grade partial tears into their calculations.

Studies examining LHB pathology in patients being treated for rotator cuff tear reported instability of the LHB in 16% to 45% of patients [8,26]. Although only 7.2% of patients in the present study were found intraoperatively to have instability of the LHB, our study focused on patients requiring arthroscopic subacromial decompression with or without rotator cuff repair and thus potentially included patients with a lesser degree of structural pathology. There is also some observer subjectivity to the characterization of LHB instability.

We found that patients with pathology of all three tendons were significantly older than patients without pathology of all three, coinciding with a study by Ogata and Uhthoff, which found that the severity and incidence of rotator cuff tears increased with age [28]. Similar to our study, Yamamoto et al. have suggested that not only does the incidence of rotator cuff tears increase with age, but it is also more likely in males and affects the dominant arm [29].

This study has several limitations. First, its retrospective design and this restriction introduce selection bias. Patients with milder pathology who responded to conservative management were not included, limiting generalizability. Second, a degree of selection bias may also be present as both physical exam and the grading of pathology were performed by a single surgeon. While this ensures consistency, it limits external reproducibility. Third, we lacked a healthy control group, which limits our ability to determine whether the observed tendon co-occurrence exceeds the baseline rates expected by chance given the confined anatomical space of the coracoacromial arch. Consequently, these findings reflect clinical associations within a surgical cohort rather than proven causal superiority over normal anatomical co-existence. Additionally, documentation of several preoperative and intraoperative variables including impingement subtype and biceps pulley involvement was incomplete in some operative reports, which may have affected the specificity of those subgroup analyses. Furthermore, because multiple pairwise chi-square tests were conducted across Tables 3-4 without a Bonferroni or similar multiplicity correction, the elevated risk of type I error means some reported associations may be false positives. Future studies with larger cohorts should employ adjusted significance thresholds or multivariate analyses to validate these coexistent tendon pathology relationships. Furthermore, because the surgeon was not blinded to the preoperative MRI findings prior to performing the arthroscopic procedures, potential observer or confirmation bias in tendon pathology grading cannot be excluded. Also, our dataset included bilateral shoulder procedures performed on 10 patients; while each shoulder was managed and analyzed as an independent clinical event due to separate symptomatic presentations, treating them as independent observations introduces a potential statistical limitation regarding complete data independence. The control group for the three-tendon pathology analysis pooled patients with zero, one, or two affected tendons, which creates a heterogeneous comparison group. While a granular dose-response trend analysis across incremental levels of tendon involvement would offer further insights, our study focused on the binary contrast of complete three-tendon failure versus less extensive disease. Despite these limitations, our study provides quantitative data on the frequency of co-involvement of the three tendons in shoulder subacromial impingement - a phenomenon often discussed anecdotally but rarely measured in a large clinical cohort.

These findings support our hypothesis that supraspinatus, subscapularis, and LHB pathologies frequently co-occur in surgically treated subacromial impingement cases. These results highlight the importance of routine systematic assessment of all three tendons during diagnostic workup and arthroscopic evaluation to ensure comprehensive identification and management of concomitant lesions.

## Conclusions

In conclusion, pathology of the supraspinatus, LHB, and subscapularis tendons frequently co-occurs in patients with subacromial impingement syndrome. Recognizing this intimate relationship highlights the clinical importance of a thorough, systematic evaluation of all three tendons to ensure complete diagnostic co-occurrence is identified during surgical exploration.
